# Supplementary material for: Causal Relationship between Aging and Anorexia Nervosa: A White-Matter-Microstructure-Mediated Mendelian Randomization Analysis
Source: Biomedicines. 2024 Aug 16;12(8):1874. doi: 10.3390/biomedicines12081874 (PMC11351342; doi:10.3390/biomedicines12081874)
Supplement: Supplementary file 1 [file biomedicines-12-01874-s001.zip › Supplementary Figure/Figure S4/Singe SNP plot of MR analysis for TL to ubm-b-1760.pdf]

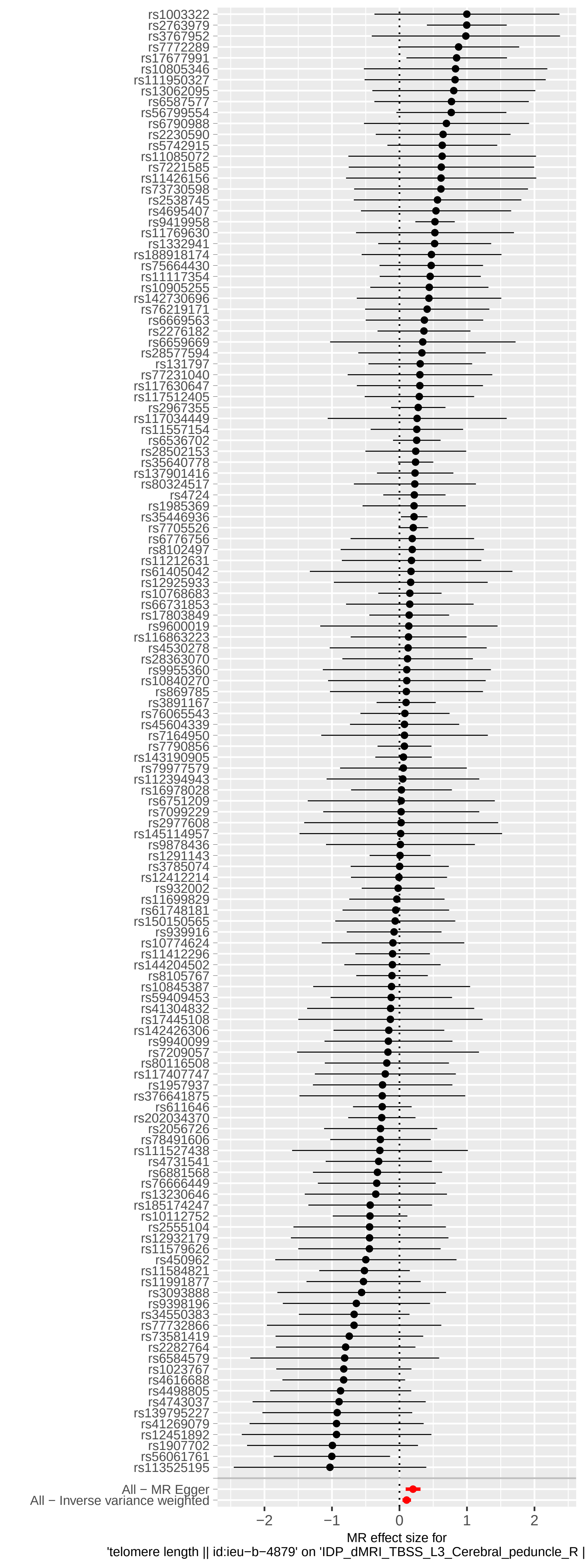
rs1003322
rs2763979
rs3767952
rs7772289
rs17677991
rs10805346
rs111950327
rs13062095
rs6587577
rs56799554
rs6790988
rs2230590
rs5742915
rs11085072
rs7221585
rs11426156
rs73730598
rs2538745
rs4695407
rs9419958
rs11769630
rs1332941
rs188918174
rs75664430
rs11117354
rs10905255
rs142730696
rs76219171
rs6669563
rs2276182
rs6659669
rs28577594
rs131797
rs77231040
rs117630647
rs117512405
rs2967355
rs117034449
rs11557154
rs6536702
rs28502153
rs35640778
rs137901416
rs80324517
rs4724
rs1985369
rs35446936
rs7705526
rs6776756
rs8102497
rs11212631
rs61405042
rs12925933
rs10768683
rs66731853
rs17803849
rs9600019
rs116863223
rs4530278
rs28363070
rs9955360
rs10840270
rs869785
rs3891167
rs76065543
rs45604339
rs7164950
rs7790856
rs143190905
rs79977579
rs112394943
rs16978028
rs6751209
rs7099229
rs2977608
rs145114957
rs9878436
rs1291143
rs3785074
rs12412214
rs932002
rs11699829
rs61748181
rs150150565
rs939916
rs10774624
rs11412296
rs144204502
rs8105767
rs10845387
rs59409453
rs41304832
rs17445108
rs142426306
rs9940099
rs7209057
rs80116508
rs117407747
rs1957937
rs376641875
rs611646
rs202034370
rs2056726
rs78491606
rs111527438
rs4731541
rs6881568
rs76666449
rs13230646
rs185174247
rs10112752
rs2555104
rs12932179
rs11579626
rs450962
rs11584821
rs11991877
rs3093888
rs9398196
rs34550383
rs77732866
rs73581419
rs2282764
rs6584579
rs1023767
rs4616688
rs4498805
rs4743037
rs139795227
rs41269079
rs12451892
rs1907702
rs56061761
rs113525195
All − MR Egger
All − Inverse variance weighted
−2
−1
0
1
2
MR effect size for
'telomere length || id:ieu−b−4879' on 'IDP_dMRI_TBSS_L3_Cerebral_peduncle_R |
